# Post-discharge body weight and neurodevelopmental outcomes among very low birth weight infants in Taiwan: A nationwide cohort study

**DOI:** 10.1371/journal.pone.0192574

**Published:** 2018-02-14

**Authors:** Chung-Ting Hsu, Chao-Huei Chen, Ming-Chih Lin, Teh-Ming Wang, Ya-Chi Hsu

**Affiliations:** 1 Division of Neonatology, Department of Pediatrics, Taichung Veterans General Hospital, Taichung, Taiwan; 2 School of Medicine, National Yang-Ming University, Taipei, Taiwan; 3 Center for Faculty Development, Taichung Veterans General Hospital, Taichung, Taiwan; 4 Department of Food and Nutrition, Providence University, Taichung, Taiwan; University of Illinois at Urbana-Champaign, UNITED STATES

## Abstract

**Background:**

Premature infants are at high risk for developmental delay and cognitive dysfunction. Besides medical conditions, growth restriction is regarded as an important risk factor for cognitive and neurodevelopmental dysfunction throughout childhood and adolescence and even into adulthood. In this study, we analyzed the relationship between post-discharge body weight and psychomotor development using a nationwide dataset.

**Materials and methods:**

This was a nationwide cohort study conducted in Taiwan. Total of 1791 premature infants born between 2007 and 2011 with a birth weight of less than 1500 g were enrolled into this multi-center study. The data were obtained from the Taiwan Premature Infant Developmental Collaborative Study Group. The growth and neurodevelopmental evaluations were performed at corrected ages of 6, 12 and 24 months. Post-discharge failure to thrive was defined as a body weight below the 3rd percentile of the standard growth curve for Taiwanese children by the corrected age.

**Results:**

The prevalence of failure to thrive was 15.8%, 16.9%, and 12.0% at corrected ages of 6, 12, and 24 months, respectively. At corrected ages of 24 months, 12.9% had low Mental Developmental Index (MDI) scores (MDI<70), 17.8% had low Psychomotor Developmental Index (PDI) scores (PDI<70), 12.7% had cerebral palsy, and 29.5% had neurodevelopmental impairment. Post-discharge failure to thrive was significantly associated with poor neurodevelopmental outcomes. After controlling for potential confounding factors (small for gestational age, extra-uterine growth retardation at discharge, cerebral palsy, gender, mild intraventricular hemorrhage, persistent pulmonary hypertension of newborn, respiratory distress syndrome, chronic lung disease, hemodynamic significant patent ductus arteriosus, necrotizing enterocolitis, surfactant use and indomethacin use), post-discharge failure to thrive remained a risk factor.

**Conclusion:**

This observational study observed the association between lower body weight at corrected age of 6, 12, and 24 months and poor neurodevelopmental outcomes among VLBW premature infants. There are many adverse factors which can influence the neurodevelopment in NICU care. More studies are needed to elucidate the causal relationship.

## Introduction

Premature infants are at high risk for developmental delay and cognitive dysfunction. [1–6] Numerous medical conditions and other variables have been shown to be associated with neurodevelopmental outcomes among premature infants, including gender, small for gestational age (SGA), intraventricular hemorrhage (IVH), periventricular leukomalacia (PVL), patent ductus arteriosus (PDA), necrotizing enterocolitis (NEC), and chronic lung disease (CLD). [4, 7–15] Furthermore, growth restriction is known to be an important risk factor for cognitive and neurodevelopmental dysfunction throughout childhood and adolescence, and even into adulthood. [1, 2, 4–6, 8, 16–24] Although early and aggressive nutritional support is a generally accepted approach for facilitating better growth in very low birth weight (VLBW) preterm infants, [25–27] post-natal growth restriction is still common among those infants. [28–30] Thus, the growth of a VLBW infant after discharge from hospitals is now considered to be an important and controllable factor for the infant’s long-term psychomotor development. [31–37] In this study, we analyzed the relationship between post-discharge body weight and psychomotor development using a nationwide dataset.

## Material and methods

This was a nationwide cohort study that was conducted in Taiwan. Premature infants born between 2007 and 2011 with a birth weight of less than 1500 g were enrolled into this study. The data were obtained from the Taiwan Premature Infant Developmental Collaborative Study Group which was established by the Premature Baby Foundation of Taiwan. The growth and neurodevelopmental evaluations were performed at corrected ages of 6, 12, and 24 months. The Bayley Scales of Infant Development-II (BSID-II) [38, 39] were used to assess neurodevelopment. The reliability and validity of BSID-II have been tested in Taiwan.[40, 41] Babies with advanced intraventricular hemorrhage (grade III and grade IV) and chromosome anomalies were excluded. Those without BSID-II scores or complete follow-up data were also excluded. All infants’ demographic data including gestational age, birth weight, gender, clinical diagnosis, co-morbidity, and follow-up outcomes after discharge were obtained from the database. The study protocol was approved by the Institutional Review Board of Taichung Veterans General Hospital. All data was accessed anonymously, and the Institutional Review Board waived the need for consent.

## Definition of post-discharge failure to thrive

Post-discharge failure to thrive (FTT) was defined as a body weight below the 3rd percentile of the standard growth curve for Taiwanese children by the corrected age. [42] The body length and head circumference measurement data may not be analyzed due to wide variations in values and much missing data during data collection.

### Primary outcomes

The primary outcome was the neurological and psychomotor performance at the corrected age of 24 months. We used outcome at corrected age of 24 months instead of 6 months and 12 months because it could be relatively regarded as a long-term outcome and was less interfered by the conditions during admission. Mental Developmental Index (MDI) score and Psychomotor Developmental Index (PDI) score were generated by BSID-II [38, 39]. A MDI/PDI score below 70 was defined as significant neurological/psychomotor impairment, and a score of 70 to 85 was defined as borderline neurological/psychomotor impairment. Cerebral palsy (CP) was defined as the presence of any of the following disorders: spastic tetraparesis, spastic hemiparesis, spastic diplegia, spastic dyskinesia, or hypotonia at corrected age of 24 months by neurologist. Visual impairment is defined as amblyopia or blindness in any eye at corrected age of 24 months by ophthalmologist. Hearing impairment is defined as more than 30 decibels(dB) hearing loss in any ear at corrected age of 24 months by otologist. All children had visual or hearing impairment would be followed by ophthalmologist or otologist. Neurodevelopmental impairment (NDI) was defined as any of the following conditions: MDI below 70, PDI below 70, CP, visual impairment, or hearing impairment. [43]

### Cofactors

We collected potential confounding factors including gender, small for gestational age (SGA), mild intraventricular hemorrhage (IVH), persistent pulmonary hypertension of newborn (PPHN), respiratory distress syndrome (RDS), chronic lung disease (CLD), hemodynamic significant patent ductus arteriosus (HSPDA), necrotizing enterocolitis (NEC), surfactant use, indomethacin use and extra-uterine growth retardation (EUGR) at discharge. SGA was defined as a birth body weight below the 10th percentile of the standard fetal growth curve. [44] CLD was defined as needing respiratory support with oxygen or positive pressure ventilation at post menstrual age of 36 weeks. HSPDA was defined as needing medical intervention or surgical ligation. EUGR was defined as a weight below the 3rd or 10th percentile of the growth curve at discharge. [45]

### Statistical analysis

The crude relative risks of post-discharge failure to thrive for low MDI score, low PDI score, CP, and NDI at corrected age of 24 months were first analyzed by univariate analysis. Then two important confounding factors, SGA and EUGR, were controlled in multiple logistic regression models (models II). Finally, all potential confounding factors were controlled in the logistic regression models (models III). The results are presented as odds ratios (OR) and 95% confidence intervals (CI). Statistical significance was defined as a p value less than 0.01. All data analysis was performing using SPSS version 22 (IBM Corporation).

## Results

A total of 4636 VLBW infants were registered in the database during the study period. Among these cases, 1752 infants who were not followed up until the corrected age of 24 months were excluded. An additional 1093 infants were excluded due to chromosome anomalies (n = 24), severe IVH (grade III ~ grade IV) (n = 360), or death (n = 709). This group of infants were not the subjects that we wanted to study. So, we did not list them in Table 1. Finally, a total of 1791 VLBW infants were enrolled in the analysis. The babies who were not followed up had a greater prevalence of NEC, RDS and CLD. However, they had less surfactant, indomethacin use, and EUGR<3%. There were no significant differences in gender, SGA, EUGR<10%, mild IVH, PPHN, significant PDA, gestational age, birth body weight, post menstrual age and body weight at discharge between these two groups. (Table 1)

**Table 1:** Demographic data.

Variables	Enrolled (N = 1791)		Not followed up (N = 1752)		P value
	N	%	N	%	
Gender					0.80
Male	934	52.2	906	51.7	
Female	857	47.9	846	48.3	
Small for gestational age	621^a^	34.8	634	36.2	0.39
EUGR at discharge(3%)	827^b^	46.5	718^a^	41.2	0.001
EUGR at discharge(10%)	1191^b^	67.0	1161^a^	66.5	0.76
Mild IVH	477^c^	27.4	530	30.3	0.06
PPHN	41^d^	2.3	44^a^	2.5	0.66
Significant PDA	603^e^	34.3	603^g^	34.4	0.92
Necrotizing enterocolitis	44^f^	2.5	78^g^	4.5	0.001
RDS	1477^g^	82.5	1516^g^	86.6	<.001
Chronic lung disease	386^h^	21.8	547^g^	31.2	<.001
Surfactant use	605	33.8	488^I^	27.9	<.001
Indomethacin use	513	28.6	222^j^	12.7	<.001
	Mean ± SD		Mean ± SD		P value
Gestational age, weeks	29.3 ± 2.7		29.5 ± 2.7		0.07
Birth body weight, g	1154.5 ± 243.0		1164.5 ± 242.2		0.22
Discharge PMA, weeks	39.2 ± 3.9		39.1 ± 3.5		0.48
Discharge weight, g	2484.5 ± 468.7		2482.1 ± 482.4		0.88

EUGR: Extra-uterine growth retardation; IVH: intraventricular hemorrhage; PDA: Patent ductus arteriosus; PMA: Post menstrual age; PPHN: Persistent pulmonary hypertension of newborn: RDS: Respiratory distress syndrome; SD: Standard deviation

Missing data:

^a^ = 7;

^b^ = 14;

^c^ = 50;

^d^ = 6;

^e^ = 32;

^f^ = 4;

^g^ = 1;

^h^ = 18;

^i^ = 2;

^j^ = 3

**Fig 1:**
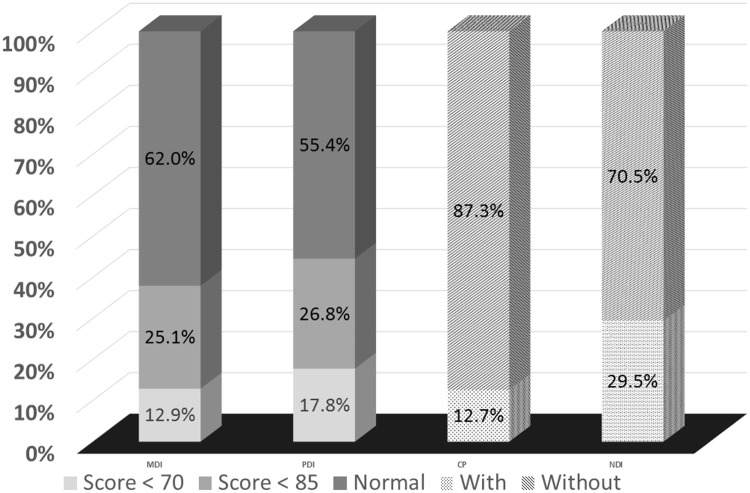
The proportions of neurodevelopmental outcomes of the enrolled infants at age of 24 months. 12.9% children had a low MDI score (MDI<70), 17.8% children had a low PDI score (PDI<70), 12.7% children had CP, and 29.5% children had NDI.

The rate of failure to thrive was 15.8% (284/1791) at the corrected age of 6 months, 16.9% (303/1791) at 12 months, and 12.0% (216/1791) at 24 months. Fig 1 showed the proportions of four different neurodevelopmental outcomes of the enrolled 1791 children at the corrected age of 24 months. Among them, 231 children (12.9%) had a low MDI score (MDI<70), 319 children (17.8%) had a low PDI score (PDI<70), 227 children (12.7%) had CP, and 528 children (29.5%) had NDI. (Fig 1)

### The association between post-discharge failure to thrive and neurodevelopmental outcomes

The post-discharge failure to thrive was significantly associated with all poor neurodevelopmental outcomes (low MDI score, low PDI score, CP, and NDI) at the corrected age of 6 months, 12 months, or 24 months by uncontrolled analysis. Odds ratio with 95% confidence intervals and p value are summarized in Table 2. Comparing with infants without FTT, the infants with FTT had higher risks of neurological and psychomotor impairment. The effects persisted since corrected age of 6 months to 24 months.

**Table 2:** Risk of poor neurodevelopmental outcome by failure to thrive at different corrected ages (uncontrolled analysis).

Outcome	FTT (+)	FTT (-)	OR	95% CI	p
At corrected age of 6 months					
MDI<85	149/284(52.5%)	501/1436(34.9%)	2.06	1.59–2.66	<.0001
MDI<70	71/284(25.0%)	147/1436(10.2%)	2.92	2.13–4.02	<.0001
PDI<85	162/284(57.0%)	600/1436(41.8%)	1.85	1.43–2.39	<.0001
PDI<70	87/284(30.6%)	218/1436(15.2%)	2.47	1.85–3.30	<.0001
CP	63/284(22.2%)	151/1436(10.5%)	2.43	1.75–3.36	<.0001
NDI	121/284(42.6%)	384/1436(26.7%)	2.03	1.56–2.65	<.0001
At corrected age of 12 months					
MDI<85	155/303(51.2%)	493/1415(34.8%)	1.96	1.53–2.52	<.0001
MDI<70	66/303(21.8%)	151/1415(10.7%)	2.33	1.69–3.21	<.0001
PDI<85	173/303(57.1%)	583/1415(41.2%)	1.90	1.48–2.44	<.0001
PDI<70	80/303(26.4%)	219/1415(15.5%)	1.96	1.46–2.63	<.0001
CP	65/303(21.5%)	152/1415(10.7%)	2.27	1.65–3.13	<.0001
NDI	125/303(41.3%)	376/1415(26.6%)	1.94	1.50–2.51	<.0001
At corrected age of 24 months					
MDI<85	115/216(53.2%)	529/1488(35.6%)	2.06	1.55–2.75	<.0001
MDI<70	57/216(26.4%)	162/1488(10.9%)	2.94	2.08–4.14	<.0001
PDI<85	135/216(62.5%)	622/1488(41.8%)	2.32	1.73–3.11	<.0001
PDI<70	74/216(34.3%)	231/1488(15.5%)	2.84	2.07–3.88	<.0001
CP	65/216(30.1%)	156/1488(10.5%)	3.68	2.63–5.14	<.0001
NDI	104/216(48.1%)	401/1488(26.9%)	2.52	1.88–3.37	<.0001

CI: Confidence interval; CP: Cerebral palsy; FTT: Failure to thrive; MDI: Mental developmental index; NDI: Neurodevelopmental impairments; PDI: Psychomotor developmental index; OR: Odds ratio

### The association between post-discharge failure to thrive and neurodevelopmental outcomes after adjusting for important confounding factors

**Fig 2:**
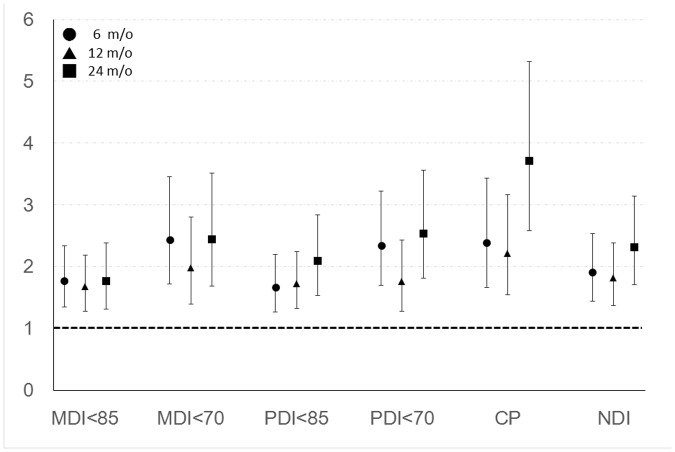
Odds ratios of post-discharge failure to thrive for poor neurodevelopmental outcomes after adjusting for small for gestational age and extra-uterine growth retardation. X-axis showed the six difficult outcomes at corrected age of 24 months, and Y-axis showed the odds ratios. ● stands for FTT at corrected age of 6 months, ▲ stands for FTT at corrected age of 12 months, and ■ stands for FTT at corrected age of 24 months. The 95% Confidence interval was presented after the odds ratio.

The numbers of infants with SGA and EUGR in both post-discharge failure to thrive and no post-discharge failure to thrive group at different corrected ages are presented in Table 3. SGA and EUGR were both significant cofactors for post-discharge failure to thrive. Then we adjusted SGA and EUGR in the multiple logistic regression models. The results were summarized in Fig 2. Post-discharge FTT at corrected age of 24 months was importantly associated with low PDI score (OR 2.54; 95% CI 1.82–3.55) and CP (OR 3.71; 95% CI 2.58–5.32) after adjusting SGA and EUGR.

**Table 3:** Risk of failure to thrive at different corrected ages by small gestational age or extra-uterine growth retardation < 3% and < 10% at discharge.

Cofactors	FTT (+)	FTT (-)	Odds ratio (95%CI)
At corrected age of 6 months, missing data:90			
SGA(+)	175/275(63.6%)	418/1426(29.3%)	4.22(3.22–5.53) ***
SGA(-)	100/275(36.4%)	1008/1426(70.7%)	
EUGR3(+)	218/275(79.3%)	577/1426(40.5%)	5.63(4.13–7.67) ***
EUGR3(-)	57/275(20.7%)	849/1426(59.5%)	
EUGR10(+)	247/275(89.8%)	892/1426(62.6%)	5.28(3.52–7.92) ***
EUGR10(-)	28/275(10.2%)	534/1426(37.4%)	
At corrected age of 12 months, missing data:94			
SGA(+)	178/292(61.0%)	412/1405(29.3%)	3.76(2.90–4.89) ***
SGA(-)	114/292(39.0%)	993/1405(70.7%)	
EUGR3(+)	218/292(74.7%)	574/1405(40.9%)	4.27(3.21–5.67) ***
EUGR3(-)	74/292(25.3%)	831/1405(59.1%)	
EUGR10(+)	255/292(87.3%)	883/1405(62.9%)	4.07(2.84–5.85) ^***^
EUGR10(-)	37/292(12.7%)	522/1405(37.2%)	
At corrected age of 24 months, missing data:106			
SGA(+)	121/209(57.9%)	463/1476(31.4%)	3.01(2.24–4.04) ***
SGA(-)	88/209(42.1%)	1013/1476(68.6%)	
EUGR3(+)	158/209(75.6%)	629/1476(42.6%)	4.17(2.99–5.82) ***
EUGR3(-)	51/209(24.4%)	847/1476(57.4%)	
EUGR10(+)	181/209(86.6%)	955/1476(64.7%)	3.53(2.34–5.32) ***
EUGR10(-)	28/209(13.4%)	521/1476(35.3%)	

CI: Confidence interval; EUGR3: Extra-uterine growth retardation < 3%; EUGR10: Extra-uterine growth retardation < 10%; FTT: Failure to thrive; SGA: Small for gestational age

*** P <.0001

**Fig 3:**
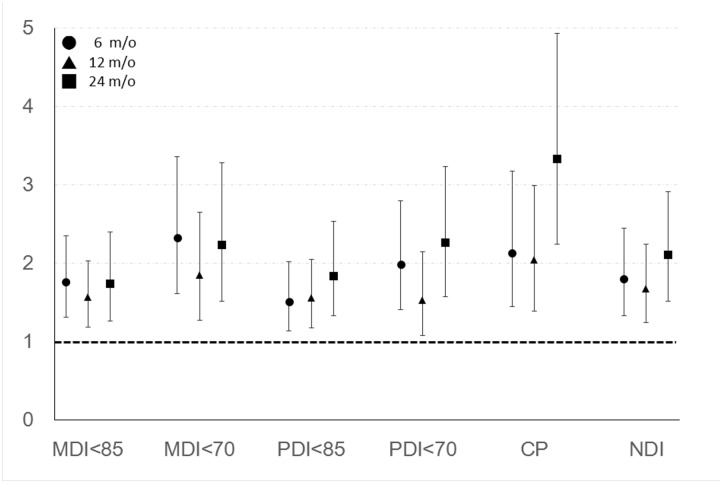
Odds ratios of post-discharge failure to thrive for poor neurodevelopmental outcomes after adjusting for all potential confounding factors. X-axis showed the six difficult outcomes at corrected age of 24 months, and Y-axis showed the odds ratios. ● stands for FTT at corrected age of 6 months, ▲ stands for FTT at corrected age of 12 months, and ■ stands for FTT at corrected age of 24 months. The 95% Confidence interval was presented after the odds ratio.

In the second model, we adjusted for all cofactors in the multiple logistic regression models. The results are summarized in Fig 3. Post-discharge FTT at corrected age of 24 months was importantly associated with CP (OR 3.33; 95% CI 2.24–4.93) after controlling all available factors.

SGA and EUGR are both significant cofactors for post-discharge failure to thrive and for poor neurodevelopment outcomes. So, we adjusted these two important cofactors in model 2 to see if their effects were independent to each other. Furthermore, because CP could be an important cofounding factor, we tried to adjust it in multiple logistic regression models. The results were summarized in Table 4. After controlling CP and SGA, FTT was still a significant risk factor for poor neurodevelopmental outcomes. (Model 3 in Table 4) The odds ratios of FTT in Model 1 (controlling SGA) and Model 2 (controlling SGA and EUGR) were not different significantly. EUGR might not be a confounding factor for the correlation between FTT and neurodevelopmental outcomes in this study.

**Table 4:** Risk of poor neurodevelopmental outcomes by failure to thrive at corrected age of 24 months.

Outcomes	Model 0		Model 1		Model 2		Model 3	
	OR	95%CI	OR	95%CI	OR	95%CI	OR	95%CI
MDI<85	2.06	1.55–2.75***	1.97	1.47–2.64***	1.82	1.35–2.46***	1.42	1.03–1.96*
MDI<70	2.94	2.08–4.14***	2.68	1.88–3.82***	2.57	1.79–3.69***	1.52	1.00–2.30*
PDI<85	2.32	1.73–3.11***	2.26	1.68–3.05***	2.10	1.55–2.84***	1.59	1.14–2.22**
PDI<70	2.84	2.07–3.88***	2.89	2.09–4.01***	2.66	1.91–3.71***	1.70	1.14–2.53**

CI: Confidence interval; MDI: Mental Developmental Index; OR: Odds ratio; PDI: Psychomotor Developmental Index

Model 0: Crude model

Model 1: Adjusted for Small for gestational age

Model 2: Adjusted for Small for gestational age and Extra-uterine growth retardation<10%

Model 3: Adjusted for Small for gestational age and Cerebral palsy

*p <.05

**p <.01

***p <.0001

## Discussion

This nationwide cohort study revealed that post-discharge failure to thrive in very low birth weight (VLBW) infants was highly associated with poor neurodevelopmental outcomes at three different time points (corrected ages of 6, 12, and 24 months). And, there is no obvious evidence showing that SGA and EUGR play important roles in the correlation between post-discharge FTT and poor neurodevelopmental outcomes.

Post-discharge failure to thrive among VLBW infants has been reported to be associated with poor psychomotor and neurodevelopmental outcomes in Western countries. [31–37] The expression of developmental delay continues throughout early childhood and even into the school-age years.[31–37] The present study was a nationwide investigation that demonstrated an associations between post-discharge failure to thrive and poor neurodevelopmental outcomes in an East Asian population. The strengths of this study include its large sample size, the use of multi-center participation, and longitudinal follow-up. Our findings provide further evidence showing the crucial importance of post-discharge nutritional support for adequate growth in VLBW infants.[25, 26, 46]

In our clinical practice, cases with significant neurological/psychomotor impairment (MDI/PDI scores<70) would receive aggressive intervention and rehabilitation. On the other hand, close observation and follow-up would be suggested for cases with borderline neurological/psychomotor impairment (MDI/PDI scores between 70 and 85). That’s the reason we estimated two levels of MDI and PDI in our study. Besides, advanced IVH (grade III and grade IV) is strongly associated with poor neurodevelopmental outcomes, but mild IVH (grade I and grade II) is still controversial.[47–49] So we excluded the cases with advanced IVH. Mild IVH was kept for analysis as a cofactor. There were no cases with PVL in the enrolled group.

SGA has been recognized as a risk factor for psychomotor retardation, speech delay, cognitive impairment, behavior problems, and poor intelligence outcome in later life.[31, 50–56] Poor growth velocity during hospitalization (EUGR) was also related to poor psychomotor outcome in early childhood.[25, 34, 43, 57, 58] Thus, early and aggressive nutritional support for adequate body weight gain during hospitalization in VLBW infants, which may mitigate the risks of neurodevelopmental disorders, appears to be the current consensus of opinion in the literature.[25, 43, 57–59] In our study, after adjusting for SGA and EUGR, failure to thrive after discharge was still an independent factor for poor neurodevelopmental outcomes in the VLBW infants. The growth status was important to the neurodevelopment of those preterm infants not only at birth and during admission, but also after discharge. Although many other factors can also predispose those infants’ growth, aggressive nutrition care and close following up growing status should be emphasized to those who take care those infants.

The Bayley scale is not an ideal test of neurodevelopment[60, 61], but the reliability and validity of BSID-II have been tested in Taiwan.[40, 41] The infants with cerebral palsy may grow differently compared with those without cerebral palsy.[62–65] So we analyzed the association between failure to thrive and neurodevelopmental outcomes at corrected age of 24 months after controlling CP. Failure to thrive was still a significant risk factor for low MDI or low PDI score.

There were certain limitations in this study. Although antenatal steroid may be an important confounding factor to the neurodevelopmental outcomes[66], there is no data in the database. Many morbidities such as low brain volume, feeding difficulties, and cortical structural abnormalities might also lead to slower growth and poor neurodevelopment outcomes.[67–70] Furthermore, events and procedures that create stress for these infants and parents in the neonatal intensive care unit might also have adverse effects to the cognitive and family development.[71] Nutrition and faster growth are not necessarily the solution to these problems. The body length and head circumference measurement data may not be reliable as wide variations in values were noted during data collection and some data were missing. As a result, we only analyzed body weight as the indicator for growth. Second, during enrollment of patients, we excluded infants without complete follow-up. This may have introduced some selection bias. The large number of loss to follow-up was almost equivalent to the final enrolled sample size. The important differences between these groups were presented in Table 1. Third, we defined EUGR as body weight at discharge below the 3rd or 10th percentile by Fenton growth curve, 2013. However, some EUGR infants may be healthy group of prematurity due to premature contraction of water spaces after birth and physiological deviation of postnatal growth trajectories.[72] Fourth, we have no data about the linear growth, nutritional status and health condition of these infants after discharge. Whether there is direct causal relationship between post-discharge nutrition and growth in these infants could not be proved in this study. Fifth, the follow-up time was only two years. Therefore, the effects in later childhood and during the school-age years could not be addressed in this study. The Taiwan Premature Infant Developmental Collaborative Study Group has extended the follow-up time to 5 years in recent years. Sixth, certain babies were lost to follow-up might limit the validity for this study.

In conclusion, this observational study observed the association between lower body weight at corrected age of 6, 12, and 24 months and poor neurodevelopmental outcomes among VLBW premature infants. There are many adverse factors which can influence the neurodevelopment in NICU care. More studies are needed to elucidate the causal relationship.

## Supporting information

S1 File Original data. (XLS)
