# Correction: Post-discharge body weight and neurodevelopmental outcomes among very low birth weight infants in Taiwan: A nationwide cohort study

**DOI:** 10.1371/journal.pone.0198310

**Published:** 2018-05-24

**Authors:** Chung-Ting Hsu, Chao-Huei Chen, Ming-Chih Lin, Teh-Ming Wang, Ya-Chi Hsu

## Notice of republication

This article was republished on May 18, 2018 to replace an incorrect version of S1 File. Please download this article again to view a corrected file.
