# Correction: Post-discharge body weight and neurodevelopmental outcomes among very low birth weight infants in Taiwan: A nationwide cohort study

**DOI:** 10.1371/journal.pone.0211526

**Published:** 2019-01-25

**Authors:** Chung-Ting Hsu, Chao-Huei Chen, Ming-Chih Lin, Teh-Ming Wang, Ya-Chi Hsu

There are errors in the Acknowledgements section. The correct acknowledgements are as follows: We acknowledge that the Premature Baby Foundation of Taiwan sponsored the Taiwan Premature Infant Developmental Collaborative Study Group. Taiwan Premature Infant Developmental Collaborative Study Group: Lead contact, Kuo-Inn Tsou (gloria0320@gmail.com); National Taiwan University Hospital, Dr. Wu-Shiun Hsieh (Director, hsiehws@ntuh.gov.tw); Mackay Memorial Hospital in Taipei, Dr. Chyong-Hsin Hsu (Director, t200441@yahoo.com.tw); Shin-Kong Wu Ho-Su Memorial Hospital, Dr. Shu-Chi Mu (Director, musc1006@yahoo.com.tw); China Medical University Hospital in Taichung, Dr. Hung-Chih Lin (Director, d0373@mail.cmuh.org.tw); Taichung Veterans General Hospital, Dr. Chao-Huei Chen (Director, joy1477@gmail. com); National Cheng Kung University Hospital at Tainan, Dr. Chao-Ching Huang (Director, huangped@mail.ncku. edu.tw); Veterans General Hospital at Kaohsiung, Dr. Kai-Sheng Hsieh (Director, kshsieh@cgmh.org.tw); and Chang Gung Memorial Hospital at Linkou, Dr. Jui-Ying Lin (reyinl@adm.cgmh.org.tw). We thank the neonatologists, research nurses and residents of the participating 22 hospitals for their help with the registration, performing follow-up program and data collection.
